# Supplementary figures and images for: QSAR-Based Drug Repurposing and RNA-Seq Metabolic Networks Highlight Treatment Opportunities for Hepatocellular Carcinoma Through Pyrimidine Starvation
Source: Cancers (Basel). 2025 Mar 6;17(5):903. doi: 10.3390/cancers17050903 (PMC11898721; doi:10.3390/cancers17050903)

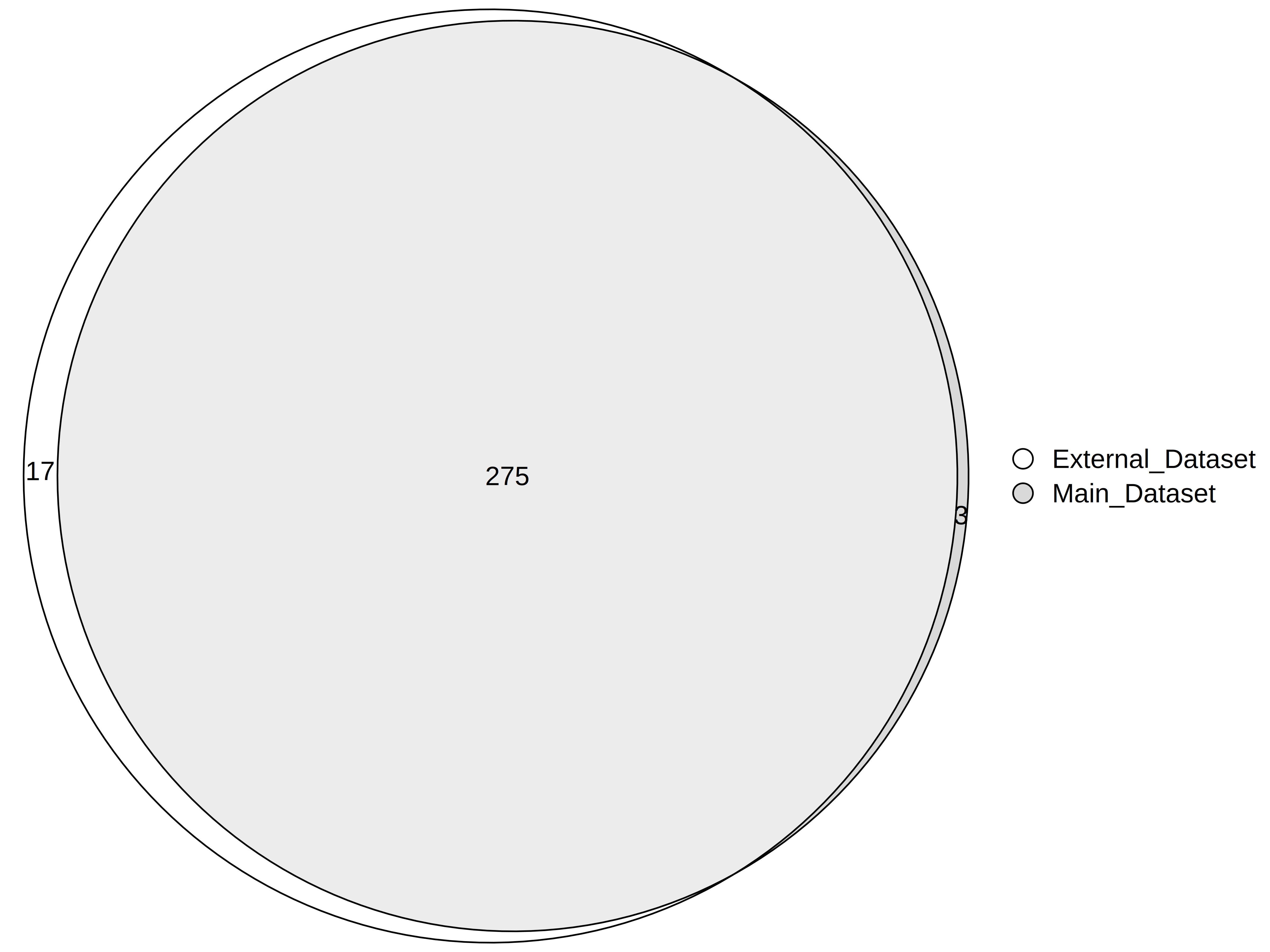

Supplement: Supplementary file 1 [file cancers-17-00903-s001.zip › Supplementary Figure S1.png]

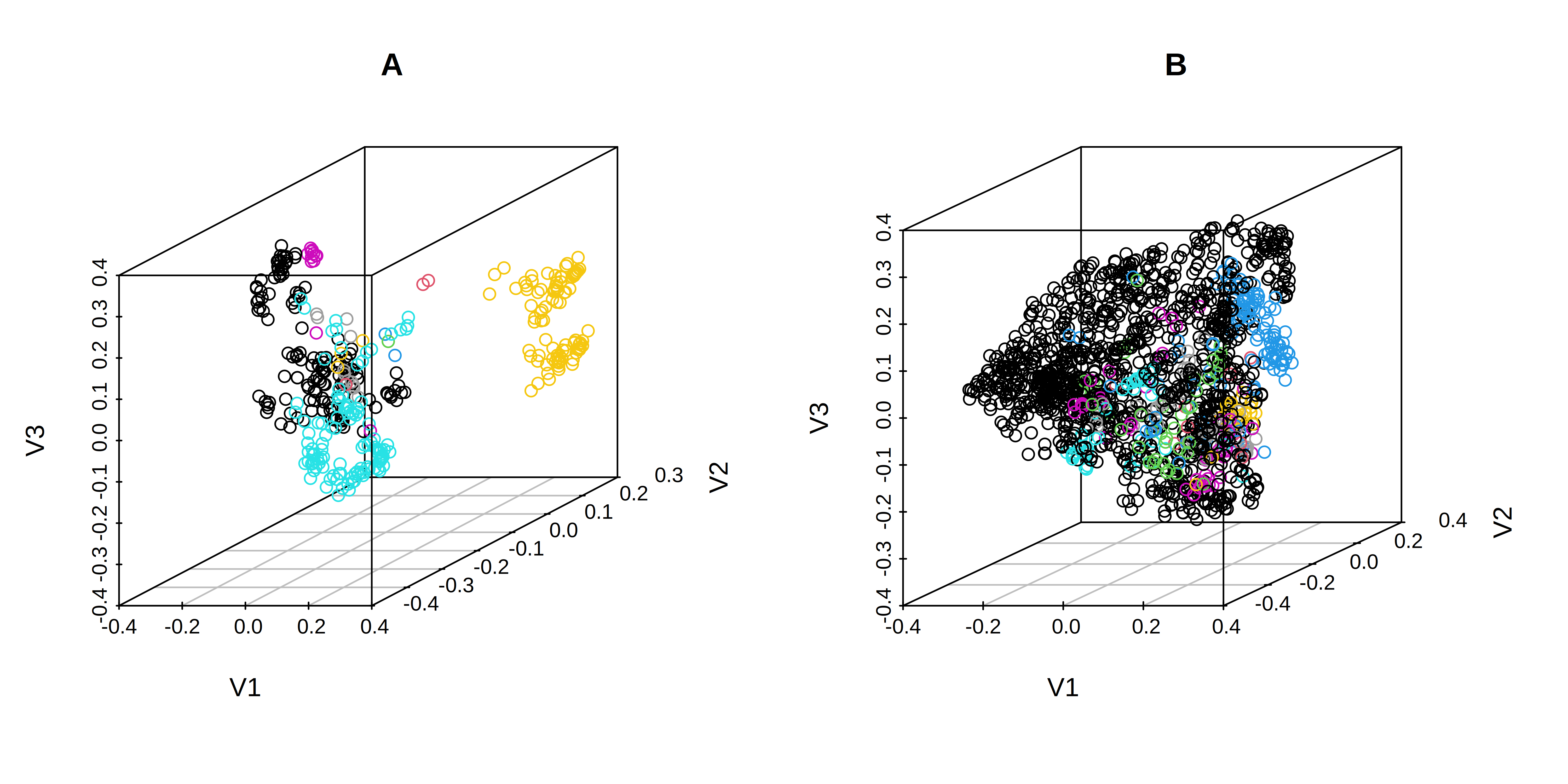

Supplement: Supplementary file 1 [file cancers-17-00903-s001.zip › Supplementary Figure S2.png]
